# A Parent-Report Gender Identity Questionnaire for Children: Psychometric Properties of an Italian Version

**DOI:** 10.1007/s10508-018-1372-7

**Published:** 2019-02-27

**Authors:** Angela M. Caldarera, Davide Marengo, Eva Gerino, Piera Brustia, Luca Rollè, Peggy T. Cohen-Kettenis

**Affiliations:** 1 Department of Psychology, University of Torino, Via Po, 14, 10123 Turin, TO Italy; 2 Department of Medical Psychology, Center of Expertise on Gender Dysphoria, EMGO Institute for Health and Care Research, VU University Medical Center, Amsterdam, The Netherlands

**Keywords:** Gender identity, Gender behavior, Gender role, Italian children

## Abstract

This article presents an Italian version of the Gender Identity Questionnaire for Children (GIQC) (Cohen-Kettenis et al., 2006; Johnson et al., 2004), a parent-report questionnaire covering a range of gender characteristics of children. We developed the GIQC-Italian version with the translation/back translation method and administered it, with a sociodemographic data sheet, to the parents of 1148 children aged 3–12 years (non-clinical sample). After obtaining descriptive data for each item, in line with Johnson et al. (2004), we examined dimensionality through exploratory factor analysis (EFA). Considering the results and that our sample was entirely non-clinical, we developed a new scoring procedure. The EFA on the new scores generated three scales: (1) a Female-Typical Behavior Scale, (2) a Male-Typical Behavior Scale, and (3) a Cross-Gender Scale. Additional EFA and confirmatory factor analyses (WLSMV estimator by using a 80/20 random-split-sample analytical approach) confirmed the three-factor solution as the best fitting dimensional structure for the revised GIQC. The Cronbach’s *α* of the scales showed a satisfactory internal consistency. The frequency distribution of the scales scores showed it is possible to find atypical gender behavior and preferences in non-clinical samples. Independent samples *t* test confirmed a significant difference between boys’ and girls’ scores. Older children reported scores indicating less gender non-conforming characteristics than younger, except for the Female-Typical Behavior Scale in the girls’ subsample. Results are discussed in the light of the existing literature about gender development. Our findings suggest that the GIQC-Italian version could be a useful tool for studying gender development in the Italian context.

**Electronic supplementary material:**

The online version of this article (10.1007/s10508-018-1372-7) contains supplementary material, which is available to authorized users.

## Introduction

For both clinical and research purposes, standardized, quantitative parent-report measures assessing gender identification, preferences, and gender behavior in children are important (Cohen-Kettenis et al., 2006). As reported by Zucker (2005), different measures of psychosexual differentiation (e.g., questionnaires, interviews, and other instruments) have been used in gender identity and gender role behavior assessment studies.

Many researchers outline that the availability of tools suitable for the measurement of gender-related constructs is essential in order to carry out screenings on community samples of children. In addition, the knowledge of normative gender-related behavior in childhood also contributes to the understanding of atypical gender development (Yu, Winter, & Xie, 2010).

Given the increasing number of children who are now clinically referred to gender identity teams in Italy (Massara, Antonelli, Mosconi, Santamaria, & Caldarera, 2014) and the customary research performed in conjunction with clinical work, there is a great need for such instruments in the Italian clinical context as well. Recently, clinicians and researchers created a national Italian Network of Gender Clinics for Children and Adolescents, with the aim of developing a shared protocol of assessment and care, in line with international guidelines, such as the Standards of Care of WPATH (Caldarera et al., 2017; Coleman et al., 2012). The integration of different techniques (e.g., standardized questionnaires, qualitative measures, clinical interviews) and sources of information (e.g., clinician’s observation, parental reports, children’s interview) is an essential requisite for a comprehensive assessment of gender variance (Zucker & Wood, 2011).

However, in Italy there is still a lack of appropriate measures and, more specifically, of a standardized questionnaire for gender-related behavior in childhood. Such a tool is needed both in a non-clinical context in order to study gender identity in Italian children and as an assessment tool for clinically referred children. To date, only two studies (Dèttore, Ristori, & Casale, 2010; Simonelli, Rossi, Tripodi, De Stasio, & Petruccelli, 2007) have been published, in which gender characteristics were measured quantitatively in a group of Italian children.

In a study on gender identity in preadolescence, Simonelli et al. (2007) used a translated version of the Gender Identity Interview for Children (GIIC) (Zucker et al., 1993) as a self-report measure for a sample of 246 children (age range: 9–13) and an adjusted Italian version of the Gender Identity Questionnaire for Children (GIQC) (Johnson et al., 2004), which was administered to the teachers of the participants. The paper presented an exploratory study, and no psychometric properties of the two measures were presented. Descriptive statistics showed that, in the examined sample, 11 (4.47%) of the children’s self-report questionnaires (adjusted Italian version of GIIC) showed non-stereotyped answers, although “no case was indicative for a potential Gender Identity Disorder (GID) as described by DSM-IV-TR” (p. 27). Regarding the modified version of the GIQC, which was administered to the teachers, 182 questionnaires were returned completed, and seven questionnaires concerning the 11 children who self-reported an atypical profile (through the GII) were collected. Out of these seven questionnaires, Simonelli et al. observed non-stereotypical behavior in four cases.

In another exploratory study, Dèttore et al. (2010) administered the GIIC to a non-clinical sample of 350 preschool children (age range 3–5), adopting the same scoring criteria defined in the original article by Zucker et al. (1993); psychometric properties of the measure were not reported. Results indicated gender-variant answers in 5.23% of the boys, 3.93% of the girls, and 4.57% of the total Italian group.

Overall, studies on Italian primary school children regarding gender development are scant. Furthermore, existing studies fail to report about the psychometric properties of the employed measures. As a result, a standardized, parent-report measure covering a broad age range (compared to only kindergarten or only pubertal age) is still missing in the Italian language.

Among parent-report questionnaires developed in other countries, the GIQC (Johnson et al., 2004) showed excellent psychometric properties in a study including both a clinical and a non-clinical group of participants. Other measures, such as the Child Game Participation Questionnaire (CGPQ) (Bates & Bentler, 1973; Meyer-Bahlburg, Sandberg, Dolezal, & Yager, 1994a) and the Child Behavior and Attitude Questionnaire (CBAQ) (Bates, Bentler, & Thompson, 1973; Meyer-Bahlburg, Sandberg, Yager, Dolezal, & Ehrhardt, 1994b), have been also used for the assessment of children’s gender preferences and behavior among school-aged children. As outlined by Johnson et al. (2004), a strength of the revised version of CBAQ (Meyer-Bahlburg et al., 1994b) was the evidence of significant normative sex differences. However, Johnson et al. also mentioned two limitations: the fact that the questionnaire was tested on children aged 6–10 (limited range) and the absence of paternal ratings (e.g., mothers as the primary informants). The CGPQ was tested on children aged 6–10 as well and is strictly related to gender-related play preferences and not gender behavior as a complex construct.

The need to create specific measures of gender behavior has been pointed out in various cultural contexts, including non-Western countries. Yu et al. (2010) reported on the Child Play Behavior and Activity Questionnaire (CPQAQ), a measure for 6–12-year-old children, based on the CBAQ and CGPQ, but with the addition 14 Chinese, gender-typical games, making it by default less convenient to be used in Italy.

Going back to the GIQC, in addition to the possibility it gives to study the phenomenon of gender variance, on such measure Johnson et al. (2004) also ran sensitivity and specificity analyses, using the scores of a clinical group which received a DSM diagnosis; the results showed good specificity and sensitivity scores in relation to DSM criteria. Moreover, the low age effects over a wide age range covering preschool to preadolescence indicated that the GIQC may be an appropriate measure to assess change over time. The GIQC has been used in other studies, including cross-national samples (Cohen-Kettenis et al., 2006). Such characteristics, along with the good psychometric properties evidenced both in clinical and in control samples, make such a questionnaire a more suitable measure for Italian children, as compared to the other cited measures. The GIQC is a 16-item questionnaire, developed by Johnson et al. (2004) as a revised version of a Gender Identity Questionnaire originally developed by Elizabeth and Green (1984). The items cover a range of gender characteristics, which Johnson et al. considered aspects of the core phenomenology of gender dysphoria, and each item was rated on a 5-point Likert scale for frequency of occurrence (three items also contain a “not applicable” option), with higher scores reflecting more gender typicality. Johnson et al. described the results of an exploratory factor analysis (EFA) performed on the maternal ratings of the GIQC on 325 gender-referred children and 504 controls from Toronto: a one-factor solution, accounting for 43.7% of the variance, was derived, including 14 of the 16 items (Items 8 and 16, with loadings < .30, were excluded). Johnson et al. presented the demographic correlates as well. The importance of testing how gender presentation varies according to different demographic characteristics is also shown by other studies conducted on samples of Italian adults (Fisher et al., 2013).

This article presents the development of an Italian version of the GIQC and the study of its psychometric characteristics. When the research design was prepared, considering that the questionnaire had to be administered to an exclusively non-clinical group (the size of the clinical group was too small when we collected the data), we bore in mind the possibility that a new coding scheme could be needed: in fact, the content of some items (see the questionnaire in Supplemental Material) of the GIQC is related to play preference and behavior (such as Item 2: “He plays with girl-type dolls, such as ‘Barbie’”), while others to gender identification (such as Item 14: “He states that he is a girl or a woman”) and to role-play (such as Item 9: ‘‘In playing ‘mother/father,’ ‘house,’ or ‘school games’, he takes the role of…”). In Johnson et al. (2004), all items were coded with higher scores reflecting more gender typicality, whether the content of the item was related to gender-typed play preference and behavior, to gender identification, or to role-play. Nonetheless, in a non-clinical group, an atypical play behavior can be present in boys and girls who do not necessarily have a cross-gender identification, and we could expect such behavior to be more frequent than cross-gender identification. Conversely, in a gender-referred group, such behaviors are mostly consistent with the phenomenology of gender dysphoria: for instance, a boy or a girl showing cross-gender play preference will likely show cross-gender identification as well. Other studies (Sunderland, Mahoney, & Andrews, 2012; Wittchen, Üstün, & Kessler, 1999) on psychometric properties of questionnaires used both in clinical and in non-clinical contexts showed that treatment-seeking clinical samples can differ significantly from community samples in response to diagnostic measures. These studies suggest that tests scoring and interpretation of scales might need to be revised for non-clinical populations, depending on the observed structure, due to the fact that clinical samples are likely to show scores with a different frequency distribution in comparison with general population samples. Other studies outlined the importance of revising the scoring system in order to allow for psychometric improvements in measures translated in different languages (Giannakopoulos et al., 2009).

In addition, other studies mentioned the appropriateness of distinguishing different dimensions such as gender-typed behavior and gender identity (Bailey, Bechtold, & Berenbaum, 2002; Pasterski et al., 2015); this was also performed in a study which used items previously combined in gender identity measures (Pasterski et al., 2015). In a study focused on a multidimensional perspective on gender identity, Egan and Perry (2001) distinguished between gender identity and gender typing in children. Studies using other measures also pointed out the complexity and multidimensionality of gender identity: Liben, Bigler, Ruble, Martin, and Powlishta (2002) developed a measure, the COAT-PM (Children’s Occupation, Activity, and Trait Personal Measure), distinguishing children’s sex typing of the self-related to the dimensions of (1) occupation, (2) activity, and (3) trait. Moreover, Yu et al., (2010) found the items related to gender identification and role-play to load on a single factor, distinct from those related to gender-typed play preference and behavior. Golombok et al. (2008) also showed that as a result of its complexity, gender development varies as a function of age and birth-assigned gender.

The aims of this study were (1) to develop an Italian version of the GIQC, (2) to explore its psychometric properties—more precisely, to examine dimensionality and internal consistency, to come to a proposal for a scoring system, and, considering that the sample was entirely non-clinical, (3) to test the following hypotheses:

### **H1**

Gender atypical behavior and play preferences may be observed more frequently than features related to cross-gender identification;

### **H2**

It is possible to find demographic variations in the GIQC scores; specifically, in line with previous studies, it is possible to find differences as a function of age, birth-assigned gender, and parental education level.

## Method

### Participants and Procedure

After receiving the approval of the University Bioethics Committee, eight teaching districts from different areas and social contexts in the North of Italy (Piedmont) were selected. The invitation to participate in the study was first proposed to the school heads and then to the parents of the children attending the schools (i.e., nursery schools, kindergartens, primary schools, junior high schools). We thus used a non-probability, self-selection sampling method, based on capacity and willingness of the parents to participate in the research. A set of questionnaires, with a cover letter, a letter with information about the study with an informed consent form, and a return envelope were given to the participants. Upon informed consent, the parents of 1148 children, aged 3–12 years, completed the questionnaires. The mean age of the children was 8.36 (SD = 2.71) for the boys (*n* = 539) and 8.52 (SD = 2.69) for the girls (*n* = 609). Maternal ratings of all the 1148 children and 726 paternal ratings (of the same group) were obtained. Mothers had a mean age of 40.84 years (SD = 5.19) and fathers of 43.76 years (SD = 6.11). Participants’ demographic characteristics are shown in Table 1.

**Table 1:** Participants’ demographic characteristics

	Boys (*n* = 539)	Girls (*n* = 609)
Age		
*M*	8.36	8.52
SD	2.71	2.69
Range	3–12	3–12

	Mothers (*n* = 1.148)	Fathers (*n* = 726)
Parent’s age		
*M*	40.84	43.76
*SD*	5.19	6.11
Range	21–56	27–67
Parent’s marital status^a^		
Both parents	1018	682
Other	130	44

^a^In line with the method used by Johnson et al. (2004), marital status was coded as “Both parents” or “Other,” with the category “Other” including the following family constellations: single parent, separated, divorced, widowed

### Measures

The questionnaires included a sociodemographic data sheet and the Italian version of the GIQC (see Appendix [Supplemental Material]), which was developed following the translation/back translation method.^1^ Using the sociodemographic data sheet, information about birth-assigned sex, age of the parent and child, and parents’ education level and marital status were collected.

More specifically, parental education was coded into eight consecutive, ordinal levels, starting from the lower (“no education”) to the higher (“Ph.D. or other postgraduate programs”). Marital status was coded as a categorical variable, according to different family constellations (“single parent,” “married,” “living together,” “widowed,” “separated,” “divorced”), and afterward recoded as binary, with the categories “Both parents” and “Other.”

### Data Analysis

Data analysis was performed using SPSS 21 and Mplus (Muthén & Muthén, 1998–2016).

In line with the procedure presented by Johnson et al. (2004), as a preliminary step, descriptive data for each item of the GIQC as a function of birth-assigned sex were obtained. Then, an EFA on the maternal ratings was performed. Analyses were performed on the paternal ratings as well.

According to the findings of this first step and considering the fact that our sample was entirely recruited in schools and not in clinical services, a new coding procedure was used, which, according to similar studies presented in the scientific literature (Meyer-Bahlburg et al., 1994b; Yu et al., 2010), could be more suitable for a non-clinical context: in fact, some items of the questionnaire (14-item version) refer to male-typical behavior (Items 3, 6–7, 11) and others to female-typical behavior (Items 2, 4–5, 10); the scoring for these items as presented by Johnson et al. (2004) was intended to indicate cisgender (score of 5) versus cross-gender behavior (score of 1), in line with the fact that the questionnaire was tested on a sample including children referred to a gender clinic where the assessment was aimed at evaluating a diagnosis of Gender Identity Disorder, according to DSM-IV-TR (APA, 2000). Considering that our study was run on an exclusively non-clinical group, as explained in Introduction, we thought it would be appropriate to look at both masculine and feminine behaviors across the whole sample, more than considering the behavior as consistent or deviating from the same-sex peers normative behavior: therefore, we scored for the “female-typical behavior” Item “5” to indicate a high frequency of female-typical behavior and “1” to indicate an absence of these behaviors; the same was carried out for the male-typical behavior items. For the remaining items, we used the scoring method presented by Johnson et al. (2004): higher scores (5) indicate gender-typical characteristics, whereas lower (1) indicate cross-gender features. The scoring for each item is shown, between parentheses, in Appendix (Supplemental Material).

A new EFA was then performed using the new GIQC scoring, and the factor structure was analyzed accordingly. In order to confirm the factor solution for the revised GIQC, we performed EFA and CFA analyses using the WLSMV estimator by using a 80/20 random-split-sample analytical approach. Reliability of the measure was tested through the Cronbach’s *α*; we checked the correlations between the scales scores obtained from maternal and paternal ratings as well.

As a final step, we tested demographic variations. We compared, by using independent samples *t* tests, the GIQC scores of the two subgroups of birth-assigned boys and birth-assigned girls; we then compared the GIQC scores of subgroups of children of different ages (divided in the three age blocks 3–5, 6–9, and 10–12). Additionally, general linear modeling was conducted to compare children in different age blocks (3–5, 6–9, 10–12) on the GIQC scores. Specifically, multivariate (MANOVA) and univariate analyses of variance (ANOVA) were used to examine hypothesis of age-related multivariate and univariate differences in the prevalence of cross-gender identification, male- and female-typical behavior. Post hoc pairwise comparisons were computed using Bonferroni correction. Analyses were performed separately on the girls’ and boys’ datasets. The association between the GIQC ratings and maternal education level was tested through a Spearman’s correlation. We tested the differences between the means of the GIQC scores as a function of marital status^2^ by using independent samples *t* test.

## Results

### Descriptive Statistics

Table 2 shows frequencies for the ratings of each item on the GIQC as a function of birth-assigned sex. The data show a similar trend in the two samples for most of the items. Some differences can be seen in Item 1, regarding the playmates preference, and Item 16 (“Talks about liking his sexual anatomy”). As expected for non-clinical samples, the data show high ratings for same-gender behavior and low for gender-variant behavior, although for Items 4 and 5 for girls and 6 and 7 for boys the ratings were less extremely distributed. Descriptive statistics and comparisons between the two groups of birth-assigned boys and girls are shown in Table 3: all items showed significant group differences, except for Items 9 and 15. Further, based on Cohen’s *d* we found these mean differences to be larger for items assessing gender-typical behaviors (Items 1–8, 10–11, *d* ≥ .85), while effect size for items assessing cross-gender features (role and dress-up play; identification) was much smaller (Items 9, 12, 13–16, *d* ranging from 0 to .21).

**Table 2:** Frequencies for each item of the presented Italian version of the GIQC

	Group	
	Boys	Girls
Item 1: Favorite playmates	*N* = 536	*N* = 606
Same-sex	42.5%	36.3%
Equal	55.6%	61.6%
Cross-sex	1.9%	2.1%
Item 2: Play with girl-type dolls	*N* = 536	*N* = 607
Frequently/favorite toy	9.5%	84.7%
Once-in-a-while	9.3%	9.6%
Very rarely/never	81.2%	5.8%
Item 3: Play with boy-type dolls	*N* = 535	*N* = 607
Frequently/favorite toy	84.9%	9.6%
Once-in-a-while	6.0%	30.3%
Very rarely/never	9.1%	60.1%
Item 4: Experiments with cosmetics and jewelry	N = 539	*N* = 607
Frequently/favorite activity	1.3%	49.3%
Once-in-a-while	3.9%	36.1%
Very rarely/never	94.8%	14.7%
Item 5: Imitates female characters on TV/movies	*N* = 535	*N* = 606
Frequently/favorite activity	2.2%	20.5%
Once-in-a-while	6.5%	30.7%
Very rarely/never	91.2%	48.8%
Item 6: Imitates male characters on TV/movies	*N* = 536	*N* = 606
Frequently/favorite activity	16.0%	3.1%
Once-in-a-while	30.8%	10.2%
Very rarely/never	53.2%	86.6%
Item 7: Plays sport with boys (but not girls)	*N* = 525	*N* = 595
Frequently/favorite activity	53.3%	11.8%
Once-in-a-while	11.8%	21.8%
Very rarely/never	34.9%	66.4%
Item 8: Plays sport with girls (but not boys)	*N* = 522	*N* = 595
Frequently/favorite activity	15.9%	51.9%
Once-in-a-while	11.9%	14.1%
Very rarely/never	72.2%	34.0%
Item 9: Roles in fantasy play	*N* = 260	*N* = 511
Same-sex	89.6%	90.0%
Equal	9.2%	7.6%
Cross-sex	1.2%	2.3%
Item 10: Plays “girl-type” games	*N* = 533	N = 605
Frequently/favorite activity	8.3%	81.3%
Once-in-a-while	5.6%	8.4%
Very rarely/never	86.2%	10.3%
Item 11: Plays “boy-type” games	*N* = 528	*N* = 609
Frequently/favorite activity	82.4%	7.6%
Once-in-a-while	6.4%	20.4%
Very rarely/never	11.2%	72.1%
Item 12: Dress-up play	*N* = 289	*N* = *300*
Same-sex	87.2%	85.3%
Equal	11.1%	9.7%
Cross-sex	1.7%	5.0%
Item 13: States wish to be opposite sex	*N* = 536	*N* = *599*
Frequently/every day	0.4%	1.3%
Once-in-a-while	0.7%	2.7%
Very rarely	2.4%	4.8%
Never	96.5%	91.2%
Item 14: States he is the opposite sex	*N* = 538	*N* = 596
Frequently/every day	0.7%	2.2%
Once-in-a-while	0.6%	1.7%
Very rarely	1.3%	2.2%
Never	97.4%	94.0%
Item 15: Talks about disliking sexual anatomy	*N* = 539	*N* = 606
Frequently/every day	0.4%	0.0%
Once-in-a-while	0.6%	1.0%
Very rarely	1.3%	1.7%
Never	97.8%	97.4%
Item 16: Talks about liking sexual anatomy	*N* = 537	*N* = 606
Frequently/every day	4.1%	3.0%
Once-in-a-while	11.0%	9.7%
Very rarely	20.9%	16.8%
Never	64.1%	70.5%

As in the original article, for Items 1, 9, and 12, the same-sex and cross-sex categories combined the response options of always and usually. For Items 2–8 and 10–11, the response options of *favorite* or *frequently* and *very rarely* or *never* were combined. For Items 13–14, the response options of *every day* and *frequently* were combined. For Items 1, 9, and 12, there was a *not applicable* option (e.g., “does not play with other children,” “does not play these games”). Variation in *N* across items reflects missing data and/or endorsement of the *not applicable* option

Maternal ratings

**Table 3:** Item mean scores for birth-assigned boys compared to birth-assigned girls (presented Italian version of the GIQC)

Item	Boys *M* (SD)	Girls *M* (SD)	*t*	Cohen’s *d*
1 Favorite playmates (5 = Boys; 1 = Girls)	3.46 (0.65)	2.62 (0.61)	22.65***	1.34
2 Play with girl-type dolls (5 = Favorite toy; 1 = Never)	1.80 (1.09)	4.14 (0.86)	− 40.53***	− 2.40
3 Play with boy-type dolls (5 = Favorite toy; 1 = Never)	4.26 (1.05)	2.32 (0.90)	33.62***	2.00
4 Experiments with cosmetics and jewelry (5 = Favorite activity; 1 = Never)	1.30 (0.62)	3.37 (0.94)	− 43.32***	− 2.57
5 Imitates female characters on TV/movies (5 = Favorite activity; 1 = Never)	1.32 (0.71)	2.51 (1.08)	− 21.71***	− 1.29
6 Imitates male characters on TV/movies (5 = Favorite activity; 1 = Never)	2.38 (1.11)	1.56 (0.81)	14.37***	0.85
7 Plays sport with boys (but not girls) (5 = Favorite activity; 1 = Never)	3.15 (1.47)	2.03 (1.12)	14.35***	0.86
8 Plays sport with girls (but not boys) (5 = Favorite activity; 1 = Never)	1.99 (1.19)	3.15 (1.46)	− 14.38***	− 0.86
9 Roles in fantasy play (5 = Same-sex; 1 = Cross-sex)	4.31 (0.70)	4.33 (0.75)	− 0.27	− 0.02
10 Plays “girl-type” games (5 = Favorite activity; 1 = Never	1.59 (1.04)	3.97 (0.99)	− 39.24**	− 2.33
11 Plays “boy-type” games (5 = Favorite activity; 1 = Never	4.10 (1.16)	2.08 (0.95)	32.23**	1.93
12 Dress-up play (Same-sex dress-up) (5 = Same-sex; 1 = Cross-sex)	4.38 (0.81)	4.43 (0.78)	− 0.89***	− 0.07
13 States wish to be opposite sex (5 = Every day; 1 = Never)	1.05 (0.33)	1.15 (0.55)	− 3.45**	− 0.21
14 States he is the opposite sex (5 = Every day; 1 = Never)	1.05 (0.37)	1.13 (0.55)	− 2.60**	− 0.15
15 Talks about disliking sexual anatomy (5 = Every day; 1 = Never)	1.04 (0.26)	1.04 (0.23)	− 0.07	0.00
16 Talks about liking sexual anatomy (5 = Every day; 1 = Never)	1.56 (0.86)	1.45 (0.79)	2.16*	0.13

Maternal ratings

Except for Items 1, 9, and 12, for the others, we used a coding with “1” indicating no occurrence (“never”) and “5” indicating high frequency of the mentioned behavior

* *p* < .05; ** *p* < .01; *** *p* < .001

We checked the correlations between maternal and paternal ratings for each item for the cases that had both the mother and the father protocols (*n* = 583). The correlations were all high, with Pearson’s *r* ranging from .47 to .90 and significant (all with *p* < .001), both considering the whole dataset and distinguishing ratings of boys from those of girls.

### Exploratory Factor Analysis Using the Original Coding Scheme

In line with the method used by Johnson et al. (2004), a principal axis factor analysis with varimax rotation was performed on the 16 items, which were scored according to the method used in the original version. The Kaiser–Meyer–Olkin (KMO) measure verified the sample adequacy for the EFA (KMO = .77), which is higher than the suggested minimum limit of .60 (Kaiser, 1974).

The EFA on the 16 items did not support a one-factor solution but instead yielded a five-factor solution (explained variance: 46%), and generated loadings under the limit of .30 for Items 8, 15, and 16. Items 9, 12, 13, and 14 clearly loaded on a common factor, while the remaining items failed to show an interpretable loading pattern (e.g., items loading on more than one-factor and/or unexpected negative loadings). When setting a forced one-factor solution, we obtained many low loadings (Table 4) as compared to the results presented by Johnson et al. (2004).

**Table 4:** Factor loadings on the Italian and original version of the GIQC

	Italian version	Original version
Item 1: Favorite playmates	.25	**.77**
Item 2: Play with girl-type dolls	**.60**	**.74**
Item 3: Play with boy-type dolls	**.56**	**.34**
Item 4: Experiments with cosmetics and jewelry	.08	**.71**
Item 5: Imitates female characters on TV/movies	.19	**.64**
Item 6: Imitates male characters on TV/movies	**.32**	**.48**
Item 7: Plays sport with boys (but not girls)	.25	**.62**
Item 8: Plays sport with girls (but not boys)	.18	.20
Item 9: Roles in fantasy play	**.61**	**.89**
Item 10: Plays “girl-type” games	**.65**	**.83**
Item 11: Plays “boy-type” games	**.65**	**.72**
Item 12: Dress-up play	**.67**	**.91**
Item 13: States wish to be opposite sex	**.58**	**.81**
Item 14: States he is the opposite sex	**.56**	**.69**
Item 15: Talks about disliking sexual anatomy	.27	**.47**
Item 16: Talks about liking sexual anatomy	− .13	.02

Extraction method: principal axis factor analysis. The loadings over the limit of .30 are bolded

We then performed another EFA, on 14 items (excluding 8 and 16, which, in the Johnson’s et al. study, presented factor loadings < .30 and thus were not included in the analyses) and setting a forced one-factor extraction, in line with the procedure used by Johnson et al. (2004). This solution accounted for 28.4% of the variance: nine items kept a factor loading over the limit of .30, but Items 1, 4, 5, 7, and 15 did not.

Because of the relatively low explained variance, and the fact that only nine items had acceptable factor loadings, we made further analyses, performing the EFA on the paternal ratings (*N* = 726). In this case, the KMO confirmed the factorability with a value of .81 as well. EFA analyses conducted on paternal ratings showed results similar to those we found with the maternal ratings.

Given these inconclusive results, and in order to further explore the dimensional structure of the Italian adaptation of the GIQC, additional analyses were performed with weighted least squares means and variance-adjusted (WLSMV) estimation and GEOMIN (orthogonal) rotation in Mplus (Muthén & Muthén, 1998–2016). An advantage of using the WLSMV estimator is the availability of model fit statistics, as well as its suitability with non-normally distributed response data. Analyses were performed on maternal ratings. Model fit was evaluated using the following fit statistics: Comparative Fit Index (CFI) and root mean square error of approximation (RMSEA). According to conventional criteria, a good fit is indicated by CFI > 0.97, and RMSEA < 0.05, CFI < 0.95, and RMSEA > 0.08 demonstrate an acceptable fit (Hooper, Coughlan, & Mullen, 2008; Kline, 2011; Schermelleh-Engell, Moosbrugger, & Mȕller, 2003). EFA was initially conducted for a range of 1–3 factor solutions. None of the solutions showed acceptable fit based on suggested cutoffs. Model fit for the one-factor solution was poor (*χ*^2^[104] = 4025.41, *p* < .01; CFI = .62; RMSEA = .18 [90% CI .18–.19]). The two- and three-factor solutions also showed sub-optimal fit (two-factor EFA: *χ*^2^[89] = 1294.29, *p* < .01; CFI = .88; RMSEA = .11 [90% CI .10–.11]; three-factor EFA: *χ*^2^[75] = 803.84, *p* < .01; CFI = .93; RMSEA = .09 [90% CI .09–.10]). In all tested solutions, Items 1 and 16 showed loadings < .30. Further increasing the number of factors, while improving model fit, did not help achieving an interpretable dimensional structure.

Overall, EFA results showed the following pattern: a subset of items assessing cross-gender identification (i.e., statements or wish of being of the opposite sex) and cross-sex dress-up and role-playing behavior consistently loaded on a common factor, while items assessing other play behavior (i.e., typical male and female play behavior) prevalently loaded on additional factors. This pattern appears to be in line with more recent findings on both clinical and non-clinical samples indicating gender identification and gender role behavior as separate independent constructs (Pasterski et al., 2015; Yu et al., 2010). Still, in our study, items pertaining to typical male and female play behavior failed to load on a common factor and instead showed a non-interpretable loading pattern. We tentatively interpreted this failure as related to the employed scoring procedure, which, as indicated by Johnson et al. (2004), rated gender role behavior according to a “gender congruence/incongruence” polarity, rather than distinguishing between male-typical and female-typical role behaviors. This scoring approach might not be appropriate for analyses in non-clinical populations in which, compared with clinical samples, the association between gender identity and role behavior is expected to be weaker (Johnson et al., 2004) and the frequency of cross-gender behavior is generally lower (Sandberg, Meyer-Bahlburg, Ehrhardt, & Yager, 1993; Van Beijsterveldt, Hudziak, & Boomsma, 2006; Zucker, Bradley, Corter, Doering, & Finegan, 1980).

### Exploratory Factor Analysis Using the New Coding Scheme

In the light of the above considerations, of the preliminary EFA results, and of similar studies presented in the scientific literature (Meyer-Bahlburg et al., 1994b; Yu et al., 2010), we examined the adequacy of an alternative scoring: items assessing gender-typed play behavior were recoded as to indicate either female-typical (Items 2, 4, 5, 8, 10) or male-typical (Items 3, 6, 7, 11) behavior, while the remaining items were scored as to assess “gender congruence/incongruence.”

The EFA was first run on the 16-item solution (maternal ratings) using principal axis factor analysis with oblimin rotation. The choice to use an oblique rotation is related to the revision of the scoring procedure for items assessing either male- or female-typical behaviors, which were expected to show a negative correlation. Unconstrained EFA yielded a three-factor solution, yet Items 1, 8, 15, and 16 presented loadings under the limit of .30. We then performed a new EFA, but excluded Items 1, 8, 15, 16, thus using a 12-item version. Results showed three factors had eigenvalues over Kaiser’s criterion of 1 (Field, 2013), explaining 67.35% of the total variance. All the items had rotated factor loadings over .40. The items clustering on each factor indicate that Factor 1 represents female-typical behavior and Factor 2 male-typical behavior, while Factor 3 seems related to cross-gender features (identification, dress-up, and role-play). The correlation between Factor 1 (female-typical behavior) and Factor 2 (male-typical behavior) was nonsignificant, *r*(600) = .01, *p* > .05; correlations between Factor 3 (cross-gender features) were negatively related with both Factor 1 (female-typical behavior), *r*(600) = − .25, *p* < .05, and Factor 3 (male-typical behavior), *r*(600) = − .46, *p* < .05.

In order to further confirm the three-factor solution as the best fitting dimensional structure for the revised GIQC, we performed EFA and CFA analyses using the WLSMV estimator by using a 80/20 random-split-sample analytical approach on maternal ratings. Results are shown in Table 5. EFA analyses with the WLSMV estimator and GEOMIN rotation also showed the three-factor solution had good model fit (χ^2^[33] = 125.64, *p* < .01; CFI = .99, RMSEA = .05 [90% CI .05–.06]). Correlation between Factor 1 (female-typical behavior) and Factor 2 (male-typical behavior) was close to zero, *r*(917) = .01, *p* > .05, while correlations between Factor 3 (cross-gender features) were negatively correlated with both Factor 1 (female-typical behavior), *r*(917) = -.21, *p* < .05 and Factor 2 (male-typical behavior), *r*(917) = − .31, *p* < .05. Coherently, CFA analyses also indicated the fit of the three-factor solution was acceptable (χ^2^[51] = 108.29, *p* < .01; CFI = .99; RMSEA = .07 [90% CI .05–.09]). In this analysis, Factor 1 (female-typical behavior) and Factor 2 (male-typical behavior) revealed a strong negative correlation, *r*(227) = − .81, *p* < .05; Factor 2 (male-typical behavior) also showed a negative correlation with Factor 3 (cross-gender features), *r*(227) = − .32, *p* < .05. Examining factors correlations separately in the boy and girl samples, we found a strong negative correlation between Factor 1 (female-typical behavior) and Factor 2 (male-typical behavior) in each sample (Girls: *r*[115] = − .54, *p* < .05; Boys: *r*[110] = − .78, *p* < .05). In turn, Factor 3 (cross-gender features) showed a negative correlation with Factor 1 (female-typical behavior) in the boy sample, *r*(110) = − .39, *p* < .05, and with Factor 2 (male-typical behavior) in the girl sample, *r*(115) = − .63, *p* < .05.

**Table 5:** EFA and CFA analyses: factor loadings on the Italian version of the GIQC—maternal ratings—new scoring procedure

	EFA						CFA		
	Principal axis factoring with oblimin rotation (*N* = 1148)			WLSMV estimation with GEOMIN rotation (*N* = 901)			WLSMV estimation with correlated factors (*N* 247)		
	Factor 1	Factor 2	Factor 3	Factor 1	Factor 2	Factor 3	Factor 1	Factor 2	Factor 3
Item 10: Plays “girl-type” games	**.64**	− .36	.03	**.54**	− .80	.06	.99		
Item 2: Play with girl-type dolls	**.62**	− .40	− .03	**.53**	− .79	.01	.91		
Item 5: Imitates female characters on TV/movies	**.77**	.13	.07	**.51**	− .55	− .04	.65		
Item 4: Experiments with cosmetics and jewelry	**.60**	− .17	− .10	**.47**	− .62	− .03	.75		
Item 6: Imitates male characters on TV/movies	.12	**.78**	− .07	.23	**.62**	− .01		.57	
Item 11: Plays “boy-type” games	− .36	**.65**	− .01	.02	**.98**	.18		.90	
Item 3: Play with boy-type dolls	− .39	**.62**	.01	− .01	**.95**	.18		.91	
Item 7: Plays sport with boys (but not girls)	− .03	**.41**	− .04	.09	**.59**	.06		.61	
Item 12: Dress-up play	.15	.07	**.75**	.05	.01	**.93**			.81
Item 9: Roles in fantasy play	.11	.03	**.69**	− .01	− .04	**.68**			.72
Item 13: States wish to be opposite sex	− .17	− .13	**.65**	− .65	− .03	**.54**			.94
Item 14: States he is the opposite sex	− .10	− .06	**.64**	− .50	.04	**.49**			.54

As regards the EFA, the loadings of the items over the limit of .40 are highlighted in bold

We performed the same analyses on the paternal ratings, which resulted in similar solutions: the 12-item rotated, unrestricted solution explained 71.59% of the total variance with three factors extracted (criterion: eigenvalue > 1), and the items clustered on the three factors (minimum loading: .44) with the same pattern as in the maternal ratings. EFA analyses with the WLSMV estimator and GEOMIN rotation also showed the three-factor solution had good model fit (*χ*^2^[33] = 73.75, *p* < .01; CFI = .99, RMSEA = .05 [90% CI .03–.06]). Correlation between factors was not significant. Further, the CFA analysis performed on paternal ratings showed the three-factor model had good fit (*χ*^2^[51] = 72.60, *p* < .05; CFI = .99; RMSEA = .04 [90% CI .01–.05]. For both EFA and CFA analyses, factor loadings patterns were similar to those obtained on maternal ratings. Based on CFA results, Factor 1 (female-typical behavior) and Factor 2 (male-typical behavior) revealed a negative correlation, *r*(143) = − .82, *p* < .05; Factor 2 (male-typical behavior) also showed a negative correlation with Factor 3 (cross-gender features, *r*(143) = − .25, *p* < .05). When analyzing separately the boy and girl samples, we found negative correlations between Factor 1 (female-typical behavior) and Factor 2 (male-typical behavior) in both samples (Girls: *r*[77] = − .40, *p* < .05; Boys: *r*[66] = − .77, *p* < .05). Further, in the girl sample, Factor 3 (cross-gender features) showed a negative correlation with Factor 2 (male-typical behavior), *r*(77) = − .65, *p* < .05. Remaining correlations were not significant.

### Reliability

According to results from factor analyses, it was possible to construct three scales: (1) Female- and (2) Male-Typical Behavior (FTB, MTB), and (3) a Cross-Gender (CG) Scale. All the scales had a satisfactory consistency: the Cronbach’s *α* for the FTB scale was of .89 both in the maternal and in paternal ratings; for the MTB was .81 in maternal and .80 in paternal ratings; for the CG was of .76 in the maternal and of .85 in the paternal ratings.

Like in the preliminary analysis of the items (in the descriptive statistics section), we checked the correlations between maternal and paternal ratings for the scales scores by using a data set including all the cases that had both the mother and the father protocols (*n* = 583). Also, for the scale scores, the correlations were all high, with a Pearson’s *r* ranging from .61 to .91, and significant (all with *p* < .001), both considering the whole dataset and distinguishing ratings of boys from those of girls.

### Demographic Correlates

Since the analyses showed the same functioning for maternal and paternal ratings, we checked the effects for sociodemographic variables by using the maternal ratings only.

Table 6 reports mean scores as a function of sex, as well results of *t* test and effect size values.

**Table 6:** GIQC mean scale score as a function of sex

	Boys			Girls			
	*M*	SD	*N*	*M*	SD	*N*	Cohen’s *d*
FTB	1.51	.69	539	3.50**	.66	608	2.95
MTB	3.47	.84	536	1.99**	.68	608	1.99
CG	4.79	.39	539	4.65**	.51	608	0.31

Absolute range of each scale: 1–5

** *p* < .001

As expected, independent samples *t* tests confirmed that boys scored higher than girls on MTB, while girl scored higher than boys on FTB. In turn, boys scored higher than girls on the CG scale, indicating that they tended to be more gender conforming than girls, with a small effect size (*d* = .31). It is worthy to note that the CG scale showed the highest mean scores and the lowest variability, when compared with the FTB and MTB scales.

The CG scale showed a modest but significant correlation with age within the whole sample, *r*(1146) = .11, *p* < .01, and in the boy sample, *r*(536) = .18 *p* < .01, but was not correlated with age in the girl sample, *r*(606) = .05, *p* = .23. As regards FTB and MTB, the results showed a different pattern in the girl and boy samples. In the girl sample, age showed a significant negative correlation with both the MTB (*r*[606] = − .12, *p* < .01) and FTB (*r*[606] = − .27, *p* < .01) scales. Instead, in the boy sample, age showed a negative correlation with FTB (*r*[536] = − .24, *p* < .01), while no significant correlation emerged with MTB (*r*[1146] = .04, *p* = .32). In the whole sample, age was negatively related with FTB (*r*[1146] = − .12, *p* < .001), while no correlation emerged with MTB (*r*[1146] = − .04, *p* = .14).

In both groups, MANOVA showed significant between-group multivariate age-related differences on the scales (Girls: Wilks’ *Λ* = .87, *F*[6, 1206] = 13.97, *p* < .001; Boys: Wilks’ *Λ* = .92, *F*[6, 1062] = 7.74, *p* < .001). However, ANOVA results showed different patterns in the girl and boy groups. Among girls, age-related differences emerged only on the FTB (*F*[2, 605] = 33.61, *p* < .001) and MTB scales (*F*[2, 605] = 4.56, *p* < .05), while differences in the CG scale scores were not significant (*F*[2, 605] = 1.06, *p* = .35). Post hoc analyses using Bonferroni correction showed the FTB to be significantly lower (*p* < .001) in the older group (10–12: *M* = 3.25, SD = .72) when compared with both the 3–5- (*M* = 3.60, SD = .56) and 6–9-year-old (*M* = 3.70, SD = .55) age groups; in turn, MTB for the 10–12 age group (*M* = 1.90, SD = .68) was significantly lower only when compared with the youngest group (3–5: *M* = 2.13, SD = .61). Among boys, age-related differences emerged only on the FTB (*F*[2, 533] = 15.67, *p* < .001) and CG scales (*F*[2, 533] = 11.35, *p* < .001), while differences in the MTB scale scores were not significant (*F*[2, 533] = 0.76, *p* = .47). Post hoc analyses using Bonferroni correction showed the FTB to be significantly lower (*p* < .001) in the older group (10–12: *M* = 1.31, SD = .55) when compared with both the 3–5- (*M* = 1.65, SD = .64) and 6–9-year-old (*M* = 1.63, SD = .75) age groups; in turn, CG for the 10–12 age group (*M* = 4.87, SD = .24, *p* < .001) and 6–9 group (*M* = 4.78, SD = .40, *p* < .05) was significantly higher when compared with the 3–5-year-olds (*M* = 4.64, SD = .56, *p* < .05).

Results of *t* test showed no significant difference between the mean values of each of the three GIQC scales as a function of maternal marital status (FTB: *t* = − .76, *p* = .45; MTB: *t* = 1.53, *p* = .13; CG: *t* = .51, *p* = .61).

Regarding maternal education level, no significant association was found with the FTB, *r*(1146) = .05, *p* = .07, and the CG, *r*(1146) = − .04, *p* = .15); the MTB showed a significant, but negligible correlation with education, *r*(1146) = − .07, *p* = .02.

## Discussion

The original 14-item one-factor solution (Johnson et al., 2004) did not fit our data, and because our sample was entirely non-clinical, we developed a new scoring procedure, which seemed to be more suitable for the general population. Johnson et al. (2004) also acknowledged the possibility that the one-factor solution emerged “because of the high correlation between cross-gender identity and cross-gender role in a sample that included a large percentage of children with potential problems in their gender identity development” (p. 113).

We also excluded two more items as compared to the study by Johnson et al. (2004): Item 1 and Item 15. In our sample, Item 1 (“His/her favorite playmates are”) presented a frequency distribution quite different from the one found by Johnson et al. (see Table 1), in which the “equal” rating (“boys and girls equally”) was much more represented in our data (55.6% of the boys and 61.6% of the girls), compared to the Toronto control group (27.9% of the boys and 31.1% of the girls). Aydt and Corsaro (2003), in a cross-cultural study, showed that, among children in an Italian preschool (compared to those in a North American one), popular boys in the school and several girls were frequently engaged in cross-sex play. Furthermore, some previous studies on non-clinical samples (Maccoby & Jacklin, 1987; Meyer-Bahlburg et al., 1994b) showed that the same-sex/cross-sex playmates choice is not necessarily linked to children’s play style preference, which may explain the low loading of the item on the Gender-Typed Behavior Scale (a scale including play style preferences items).

Regarding Item 15 (related to verbalized anatomic dysphoria), Johnson et al. (2004) referred to it as a “relatively crude attempt to index this component of the DSM criteria. Although the mothers judge its presence to be more common among the probands than the controls […], the majority rated it as ‘never’ occurring” (p. 113). Johnson et al. also suggested that the assessment of anatomic dysphoria would possibly need a more comprehensive approach than a single item explicitly stating it: in this direction, they proposed the utility of working to create a systematic structured interview schedule, with both parents and children, focused on various indicators of anatomic dysphoria.

The results of the second EFA, showing a three-factor solution, with two factors related to male- and female-typical behavior, and one related to a cross-gender dimension, were in line with the findings of the study of Yu et al. (2010), performed on the CPBAQ in a non-clinical sample.

As regards the first hypothesis discussed in aim 3, in the present study, based on parent report, features related to cross-gender identification appeared to be less frequent than gender atypical behavior and play preferences. For example, 1.1% of the boy sample and 4.0% of the girl sample reported stating once-in-a-while or frequently of wishing to be of the opposite sex (Item 13). Instead, 13.9% of the boys and 28.0% of the girls reported occasional or frequent playing with gender atypical games (Items 10–11). Similar results were reported also by Johnson et al. (2004, see Table 1) and in studies from the USA (Achenbach & Rescorla, 2001), the Netherlands (Van Beijsterveldt et al., 2006; Verhulst, Van der Ende, & Koot, 1996), and Italy, as mentioned by Dèttore et al. (2010) in relation to the study on the Italian standardization of CBCL (Frigerio et al., 2004).

We also tested the second hypothesis mentioned in the third aim, related to demographic variations in the GIQC scale scores. For both the MTB, FTB, and the CG subscales, boys and girls scored significantly different, with girls reporting scores indicating lower levels of gender conformity compared to boys. This means that, in our sample, girls, on average, showed more gender-variant behavior compared to boys. This corresponds with the observation that, in most Western societies, a tomboy girl is much more accepted than a boy showing feminine behavior (Feinman, 1981; Lee & Troop-Gordon, 2011).

In line with the results by Johnson et al. (2004), we found that scores tend to become less gender variant with increasing age, although this effect does not seem to follow a linear trend and is generally more pronounced among boys. Thus, especially among boys, it appears that children tend to become more gender typical as age increases and they enter puberty. This tendency of the scores to indicate less gender-variant behaviors as age increases is consistent with what Bussey and Bandura (1999) remarked on: that children, in the process of growing up, get more involved in the social world and thus are more subject to social influences. Such range of social influences includes peer interactions, media representation of gender roles, and educational practices, all making them more aware of social sanctions for non-normative behavior. Still, concerning girls, we found female-typical behaviors to be less frequent among those in the 10–12-year-old age group compared with the younger groups. This result appears to be in contrast to the findings indicating the tendency of gender identification to become stronger with age (de Vries, Kreukels, Steensma, & McGuire, 2014). However, it appears to be in line with previous research, showing that girls tend to become less involved in sex-typed activities as they reach early adolescence (McHale, Shanahan, Updegraff, Crouter, & Booth, 2004). Additionally, this result appears to be in line with previous findings, highlighting the tendency for girls to show greater variation in sex-typed behavior than boys due to differences in the social pressure experienced by boys and girls to conform to gender-typical behaviors (Blakemore, Berenbaum, & Liben, 2009; Golombok, Rust, Zervoulis, Golding, & Hines, 2012).

In addition, it is important to take into account the GIQC items content: many of them refer to play behavior. As girls mature earlier than boys, their lowered scores may just reflect a decreased interest in certain types of play. In a study testing the change of children’s leisure activity preferences from the age of 5 to the age of 13, Cherney and London (2006) found that while boys’ preferences for gender-stereotyped toys did not change significantly, “girls’ interest in play with gender-stereotyped toys decreased as they grew older” (p. 722).

This study was the first attempt of adapting a parent-report gender questionnaire for 3–12-year Italian children. The result is a reliable scale, measuring (1) gender-typed play preferences and behavior and (2) cross-gender features, which can be useful for clinicians and researchers.

However, we have to take into consideration some limitations: the study was run on a non-probability sample from the general population, since, in Italy, the number of children referred to specialized, gender identity clinics is still low (the services are relatively new). When a large enough gender-referred sample becomes available, the psychometric properties of the Italian version of the GIQC should be checked again and sensitivity and specificity of the tool should be tested, just like Johnson et al. (2004) did. Regarding psychometric properties, discriminant validity and concurrent validity have not yet been examined.

Moreover, this study focused only on parental ratings, without comparing them with other sources of information, such as children’s interviews, behavior observations, or teachers’ ratings. Future research should complete this aspect. Our results raise three more issues to be analyzed in future studies.

Firstly, in Johnson et al. (2004), Item 1 (on playmate preference) showed a strong correlation with the underlying factor and was included in the final factor solution. It would be interesting to know whether it is cultural differences or other factors that resulted in the divergence in findings. Secondly, the decrease in the MTB and FTB scores among girls over the age of 10 needs to be further explored, for instance, by examining possible links with other traits like social desirability, parental attitudes, or peer relations, or also by using a longitudinal design. Finally, as we did not investigate cross-cultural factors, it remains unclear to what extent the factor structure differs from the one presented by Johnson et al. (2004) because of cultural differences. Therefore, a cross-cultural study, examining the factor structure in both a clinical and a non-clinical group, would help to achieve a better understanding of this issue.

Although these issues still have to be explored further, this study showed satisfactory psychometric properties of the Italian version of the GIQC. Furthermore, it made general population scores available, making it possible to (1) develop new studies about gender development in Italy and (2) to make comparisons with data sets from other countries.

## Electronic supplementary material

Below is the link to the electronic supplementary material.

Supplementary material 1 (PDF 68 kb)

## Footnotes

1 The Italian version is available from the corresponding author upon request.

2 In line with the method used by Johnson et al. (2004), marital status was coded as “Both parents” or “Other,” with the category “Other” including the following family constellations: single parent, separated, divorced, widowed.
