# Supplementary material for: A Parent-Report Gender Identity Questionnaire for Children: Psychometric Properties of an Italian Version
Source: Arch Sex Behav. 2019 Feb 27;48(5):1603–15. doi: 10.1007/s10508-018-1372-7 (PMC6594981; doi:10.1007/s10508-018-1372-7)
Supplement: Supplementary file 1 — Supplementary material 1 (PDF 68 kb) [file 10508_2018_1372_MOESM1_ESM.pdf]

GENDER IDENTITY QUESTIONNAIRE FOR CHILDEN – BOY VERSION – ITALIAN VERSION ADAPTED SCORING WEIGHTS IN PARENTHESES

1. His favourite playmates are
   a. always boys (5)
   b. usually boys (4)
   c. boys and girls equally (3)
   d. usually girls (2)
   e. always girls (1)
   f. does not play with other children

2. He plays with girl-type dolls, such as "Barbie"
   a. as a favourite toy (5)
   b. frequently (4)
   c. once-in-a-while (3)
   d. rarely (2)
   e. never (1)

3. He plays with boy-type dolls, such as "G.I. Joe" or "Ken"
   a. as a favourite toy (5)
   b. frequently (4)
   c. once-in-a-while (3)
   d. rarely (2)
   e. never (1)

4. He experiments with cosmetics (makeup) and jewellery
   a. as a favourite activity (5)
   b. frequently (4)
   c. once-in-a-while (3)
   d. rarely (2)
   e. never (1)

5. He imitates female characters seen on TV or in the movies
   a. as a favourite activity (5)
   b. frequently (4)
   c. once-in-a-while (3)
   d. rarely (2)
   e. never (1)

6. He imitates male characters seen on TV or in the movies
   a. as a favourite activity (5)
   b. frequently (4)
   c. once-in-a-while (3)
   d. rarely (2)
   e. never (1)

7. He plays sports with boys (but not girls)
   a. as a favourite activity (5)
   b. frequently (4)
   c. once-in-a-while (3)
   d. rarely (2)
   e. never (1)

8. He plays sports with girls (but not boys)
   a. as a favourite activity (5)
   b. frequently (4)
   c. once-in-a-while (3)
   d. rarely (2)
   e. never (1)

9. In playing "mother/father," "house," or "school" games, he takes the role of
   a. a girl or woman at all times (1)
   b. usually a girl or woman (2)
   c. half the time a girl or woman and half the time
   a boy or man (3)
   d. usually a boy or man (4)
   e. a boy or man at all times (5)
   f. does not play these games

10. He plays "girl-type" games (as compared to "boy-type" games)
    a. as a favourite activity (5)
    b. frequently (4)
    c. once-in-a-while (3)
    d. rarely (2)
    e. never (1)

11. He plays "boy-type" games (as compared to "girl-type" games)
    a. as a favourite activity (5)
    b. frequently (4)
    c. once-in-a-while (3)
    d. rarely (2)
    e. never (1)

12. In dress-up games, he likes to dress up
   a. a girl or woman at all times (1)
   b. usually a girl or woman (2)
   c. half the time a girl or woman and half the time
   a boy or man (3)
   d. usually a boy or man (4)
   e. a boy or man at all times (5)
   f. does not play these games

13. He states the wish to be a girl or a woman
   a. every day (1)
   b. frequently (2)
   c. once-in-a-while (3)
   d. rarely (4)
   e. never (5)

14. He states that he is a girl or a woman
   a. every day (1)
   b. frequently (2)
   c. once-in-a-while (3)
   d. rarely (4)
   e. never (5)

15. He talks about not liking his sexual anatomy (private
parts)
   a. every day (1)
   b. frequently (2)
   c. once-in-a-while (3)
   d. rarely (4)
   e. never (5)

16. He talks about liking his sexual anatomy (private
parts)
   a. every day (5)
   b. frequently (4)
   c. once-in-a-while (3)
   d. rarely (2)
   e. never (1)

Note 1. With the appropriate pronoun changes, the Girl Version is identical to the Boy Version.

Note 2. Score calculation: “Cross-Gender” Scale: mean of the scores of items 9, 12, 13 and 14. “Female-Typical Behavior” scale: mean of the scores of items 2 – 4 – 5 – 10. “Male-Typical Behavior” scale: mean of the scores of items 3, 6, 7, 11.

Note 3. The Italian version is available from the corresponding author upon request.
